# The primary cilium-autophagy axis in vascular homeostasis and cardiovascular disease: mechanistic crosstalk and evidence boundaries

**DOI:** 10.3389/fcvm.2026.1929329

**Published:** 2026-08-11

**Authors:** Xiaoyu Fu, Wenzhe Zhou, Chenyu Zhang, Yunpeng Bai

**Affiliations:** 1 Department of Cardiovascular Surgery, Chest Hospital, Tianjin University, Tianjin, China; 2 Department of Urology, Tianjin Institute of Urology, The Second Hospital of Tianjin Medical University, Tianjin, China; 3 Clinical School of Thoracic, Tianjin Medical University, Tianjin, China

**Keywords:** aortic aneurysm, atherosclerosis, autophagy, cardiovascular disease, hypertension, primary cilium, vascular disease

## Abstract

Primary cilia are microtubule-based sensory organelles located on the cell surface and function as cellular signaling antennae for mechanical and chemical cues. In the vascular endothelium, they participate in shear-stress sensing, Ca^2+^ signal transduction, eNOS activity regulation and vascular homeostasis. Autophagy is a conserved lysosome-dependent degradative pathway that maintains cardiovascular cell homeostasis by removing damaged organelles, regulating lipid metabolism, restraining inflammatory activation and preserving proteostasis. In this review, we discuss the bidirectional regulatory relationship between primary cilia and autophagy. Primary cilia may influence autophagic activity through Ca^2+^/AMPK/mTOR, Hedgehog and PI3KC2*α*-related pathways, whereas autophagy can reciprocally regulate ciliary homeostasis through cilia-related proteins such as OFD1 and IFT20. This cilium-autophagy interface may contribute to hypertension, atherosclerosis and aortic aneurysm by modulating endothelial mechanosensing, mitochondrial quality control, lipid handling, ROS homeostasis and inflammatory responses. Because direct evidence for causal cilium-autophagy crosstalk in the cardiovascular system remains limited, we distinguish established mechanisms from biologically plausible extrapolations and disease-associated observations. By integrating evidence from ciliary mechanosensing, autophagy regulation and vascular pathological remodeling, this review proposes the primary cilium-autophag*y* axis as a conceptual framework linking hemodynamic disturbance, organelle stress and vascular disease progression, and provides a basis for refining mechanistic hypotheses and potential therapeutic targets.

## 1 Introduction

Cardiovascular diseases (CVDs) remain a leading cause of death and disease burden worldwide (1–4). Their initiation and progression involve hemodynamic abnormalities, endothelial dysfunction, dysregulated lipid metabolism, oxidative stress, inflammation and organelle homeostatic imbalance (5). In this review, we focus primarily on vascular components of CVDs, including hypertension, atherosclerosis and aortic aneurysm, because the evidence linking primary cilia and autophagy is most directly related to hemodynamic sensing, endothelial homeostasis and vascular wall remodeling in these settings. Unless otherwise specified, the term “vascular” in this review refers primarily to the blood vasculature, because most of the available evidence is derived from vascular endothelial cells, VSMCs, macrophages and blood-vessel disease models. Whether the proposed primary cilium–autophagy mechanisms also operate in the lymphatic vasculature remains unclear owing to the current lack of direct evidence. Vascular endothelial primary cilia are enriched or altered in regions of low shear stress or disturbed flow and act as mechanosensory structures involved in shear-stress sensing, Ca^2+^ signal transduction and nitric oxide (NO) generation (6–9). Autophagy regulates mitochondrial quality control, lipid handling, inflammatory responses and proteostasis in endothelial cells, macrophages and vascular smooth muscle cells (VSMCs) (10–15).

Accumulating evidence suggests that both primary cilia and autophagy are closely associated with vascular homeostasis (16). Structural or functional abnormalities of primary cilia may weaken endothelial responses to flow stimulation and affect eNOS activity, NO generation and inflammatory activation (7, 8, 17). Autophagy dysfunction can impair lipid clearance, promote mitochondrial injury, increase ROS accumulation and amplify inflammation, thereby contributing to vascular wall remodeling, plaque progression and plaque instability (11, 12, 18, 19). These two systems may therefore participate in vascular disease progression from complementary levels: extracellular and mechanical sensing on the one hand, and intracellular quality control on the other.

However, the roles of primary cilia and autophagy in vascular disease are neither unidirectional nor fixed. Ciliary function is influenced by flow pattern, vascular region and disease stage (9). Enrichment of endothelial cilia in low-shear or disturbed-flow regions suggests a relationship with vascular lesion-prone areas, but their precise roles in mature vascular homeostasis and pathological remodeling remain incompletely defined (6, 8, 20). Autophagy is also highly cell type- and stage-dependent. Moderate autophagy generally helps remove damaged organelles, restrain inflammatory responses and maintain vascular cell homeostasis, whereas insufficient autophagy or dysregulated activation may promote endothelial injury, VSMC death, vascular calcification or plaque instability (10–12, 15, 18). Thus, neither primary cilia nor autophagy should be interpreted as uniformly protective or uniformly deleterious across complex vascular pathological settings.

More importantly, although reciprocal regulation between primary cilia and autophagy has been reported in several cellular models, cardiovascular-specific evidence remains limited. On the one hand, primary cilia may influence autophagic activity through Hedgehog signaling, Ca^2+^-related pathways or the AMPK/mTOR axis (21–26). On the other hand, autophagy can regulate the stability of cilia-related proteins such as OFD1 and IFT20, or affect ciliogenesis, ciliary length and ciliary function through molecules such as PI3KC2*α* and ATG16L1 (22, 23, 27, 28). In this review, the term primary cilium-autophag*y* axis is used in a graded sense. It includes direct crosstalk, in which manipulation of cilia alters autophagic activity or manipulation of autophagy alters ciliary structure or function; mechanistic convergence, in which both systems meet at shared nodes such as AMPK-mTOR, PI3KC2*α*-PI3P, Ca^2+^ or ROS; and disease-associated coexistence, in which ciliary abnormalities and autophagy dysfunction occur in the same pathological context without proven causality. This distinction is essential to avoid overinterpreting associative findings as established causal pathways.

### 1.1 Core questions

Against this background, this review addresses three questions. First, how do primary cilia convert mechanical, metabolic and redox cues into changes in autophagic activity or autophagic flux? Second, how does autophagy reciprocally influence ciliogenesis, ciliary length maintenance and signaling function? Third, how strong is the evidence for the primary cilium-autophagy interface in hypertension, atherosclerosis and aortic aneurysm, and where are the key mechanistic gaps?.

## 2 Primary cilia in cardiovascular cells

### 2.1 Basic structure of primary cilia

**Figure 1:**
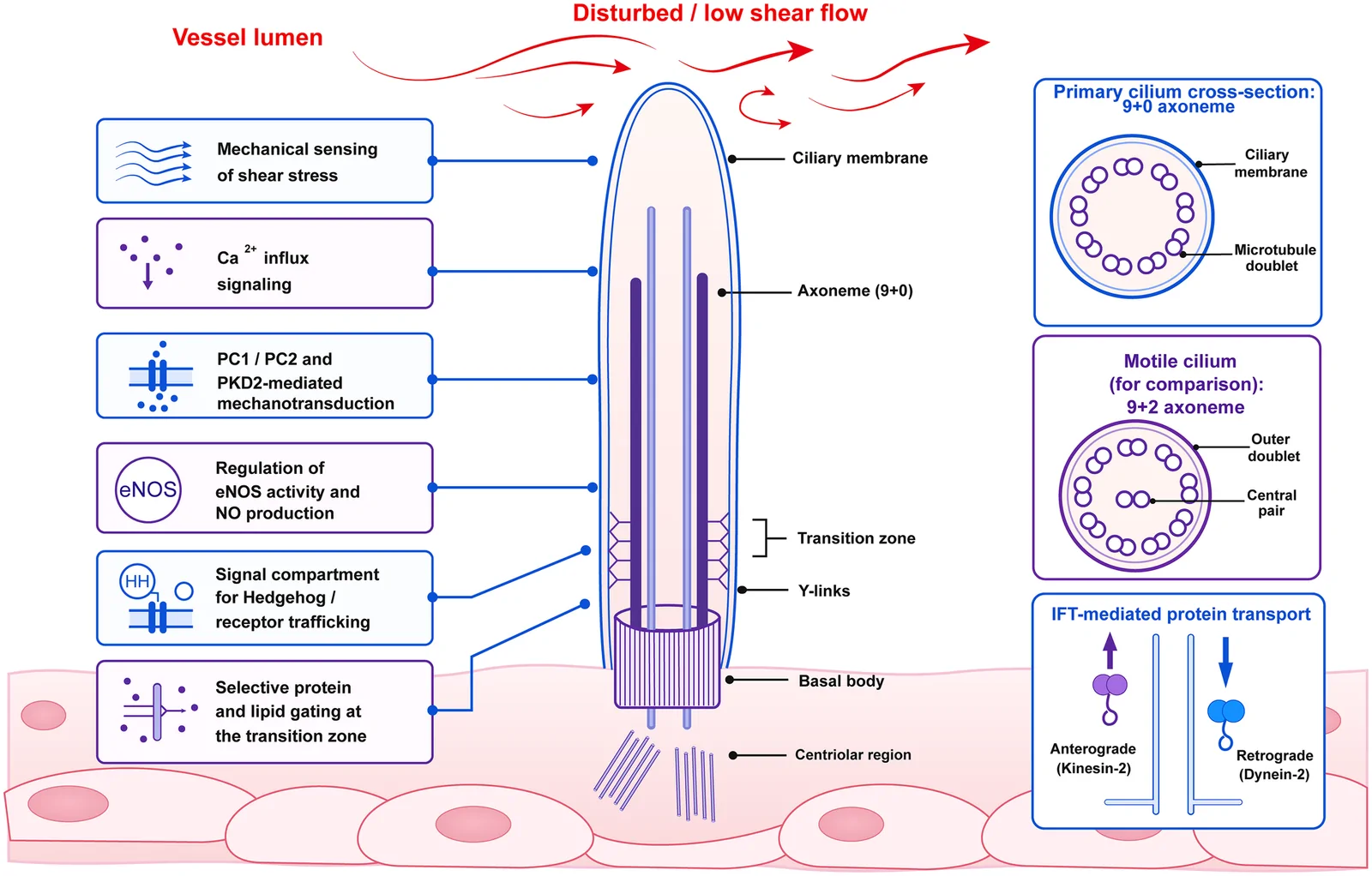
Structural organization and mechanosensory functions of primary cilia in vascular endothelial cells. Primary cilia are microtubule-based sensory organelles protruding from the apical surface of vascular endothelial cells into the vessel lumen. Under disturbed or low shear flow, endothelial primary cilia function as mechanosensory hubs that convert hemodynamic stimuli into intracellular signaling events. The schematic illustrates major ciliary components, including the ciliary membrane, 9 + 0 axoneme, transition zone, Y-link structures, basal body and centriolar region, as well as shear-stress sensing, Ca2+ influx, PC1/PC2- and PKD2-dependent mechanotransduction, eNOS/NO regulation, Hedgehog/receptor trafficking and selective protein-lipid gating. The right panels compare the 9 + 0 architecture of primary cilia with the 9 + 2 axoneme of motile cilia and show bidirectional IFT-mediated transport.

Primary cilia are non-motile sensory organelles widely present on the surface of vertebrate cells. They integrate mechanical forces, chemical ligands and local metabolic signals and convert extracellular stimuli into intracellular signaling responses (29, 30). As illustrated in Figure 1, their structural framework consists of the basal body, axoneme, transition zone (TZ) and ciliary membrane. The basal body is derived from the mother centriole and serves as the anchoring and assembly site for the primary cilium. The axoneme is composed of microtubules and extends outward to form the ciliary shaft (31). The cilium is enclosed by a ciliary membrane that is continuous with the plasma membrane but has a specialized molecular composition, allowing enrichment of specific receptors, ion channels and signaling molecules (29, 32, 33).

The transition zone lies between the basal body and the axoneme and is essential for maintaining ciliary compartmentalization (31, 34, 35). This region contains characteristic Y-link structures that form a selective barrier controlling the entry and exit of proteins, lipids and signaling molecules between the cytoplasm and the cilium (36). Classical primary cilia usually display a “9 + 0” microtubule arrangement, consisting of nine peripheral microtubule doublets without a central pair or dynein arms, and therefore generally lack active motility (29, 32, 37, 38). It should be noted that axonemal composition, ciliary length and ciliary membrane proteome vary across cell types, and such structural and molecular heterogeneity may determine the signaling capacity and functional properties of primary cilia in different tissues (32, 39, 40).

### 2.2 Functions of vascular endothelial cilia

In the cardiovascular system, primary cilia on vascular endothelial cells are primarily regarded as mechanosensory organelles (41, 42). Endothelial cilia are preferentially located in regions of low shear stress, oscillatory shear stress or disturbed flow, such as arterial branches, curvatures and recirculation zones, whereas they are less abundant or disassembled under stable laminar and high-shear conditions (6, 8, 9). Flow stimulation bends the cilium and, through polycystin complexes including polycystin-1 (PC1) and polycystin-2 (PC2/PKD2), induces Ca^2+^ signaling changes that further regulate eNOS activity and NO production (8, 9). This process is closely associated with vasodilation, endothelial barrier maintenance and anti-inflammatory responses.

When ciliary structure or function is impaired, endothelial adaptation to hemodynamic stimuli may be weakened (9). Studies have shown that loss of endothelial cilia is associated with reduced eNOS activity, increased expression of inflammation-related genes and accelerated atherosclerosis under a high-fat diet (43, 44). Ciliary loss may also influence shear-induced endothelial phenotypic transition, providing mechanistic clues for the link between abnormal flow and vascular pathological remodeling (9, 45). Primary cilia may therefore connect hemodynamic forces with endothelial signaling and contribute to vascular homeostasis and the formation of lesion-prone regions. Nonetheless, it remains important to distinguish the roles of cilia during vascular development, mature vascular homeostasis and pathological remodeling.

The formation and maintenance of primary cilia depend on the intraflagellar transport (IFT) system and multiple ciliary proteins. IFT88 is a key component of the IFT-B complex and is essential for ciliary assembly and maintenance. Endothelial-specific IFT88-deficient models suggest that endothelial cilia are not indispensable for mammalian vascular development, but their loss increases susceptibility to atherosclerosis and enhances pro-inflammatory signaling (32, 43, 46, 47). The PC1/PC2 complex represents an important molecular node linking ciliary mechanosensing, Ca^2+^ signaling and NO generation (8, 48–50). Beyond IFT and polycystins, the BBSome complex mediates ciliary membrane protein trafficking, and its dysfunction in Bardet-Biedl syndrome and related experimental models is associated with obesity, hypertension and metabolic cardiovascular risk phenotypes (51). Dopamine receptor type 5 (DR5) has also been reported to localize to cilia in vascular endothelial and renal epithelial cells and to influence ciliary length and mechanosensitivity (52). Recent genetic evidence further suggests that CDKL1 variants may contribute to thoracic aortic aneurysm susceptibility by affecting ciliogenesis and vascular wall cell function, although this finding requires validation in additional independent cohorts and cardiovascular-specific mechanistic studies (53).

Beyond mechanosensing, primary cilia regulate Hedgehog, Wnt, PDGFRalpha, mTOR and PI3K-related signaling. Smoothened (SMO), a key component of the Hedgehog pathway, must translocate to the ciliary membrane for efficient downstream activation (54). Wnt, PDGFRalpha, mTOR, PI3K and GPCR-related signals can also be modulated by ciliary structure and ciliary membrane composition (29, 55, 56). Recent studies indicate that ciliary membrane lipids, the transition-zone barrier and ciliary protein transport systems jointly shape cellular responses to external stimuli (57–59). In CVDs, the importance of primary cilia is therefore not limited to blood-flow sensing; primary cilia may also integrate Ca^2+^, Hedgehog, AMPK/mTOR, PI3K, ROS and inflammatory pathways and provide a structural basis for crosstalk with autophagy and metabolic homeostasis (8, 17, 29, 43, 60, 61).

These structural features allow endothelial primary cilia to act as compartmentalized mechanosensory organelles that couple disturbed-flow sensing with Ca²⁺ signalling, PC1/PC2–PKD2-dependent mechanotransduction, eNOS activation and selective protein/lipid gating at the transition zone (Figure 1).

## 3 Autophagy in cardiovascular homeostasis

### 3.1 Basic autophagy pathways and core regulatory axes

**Figure 2:**
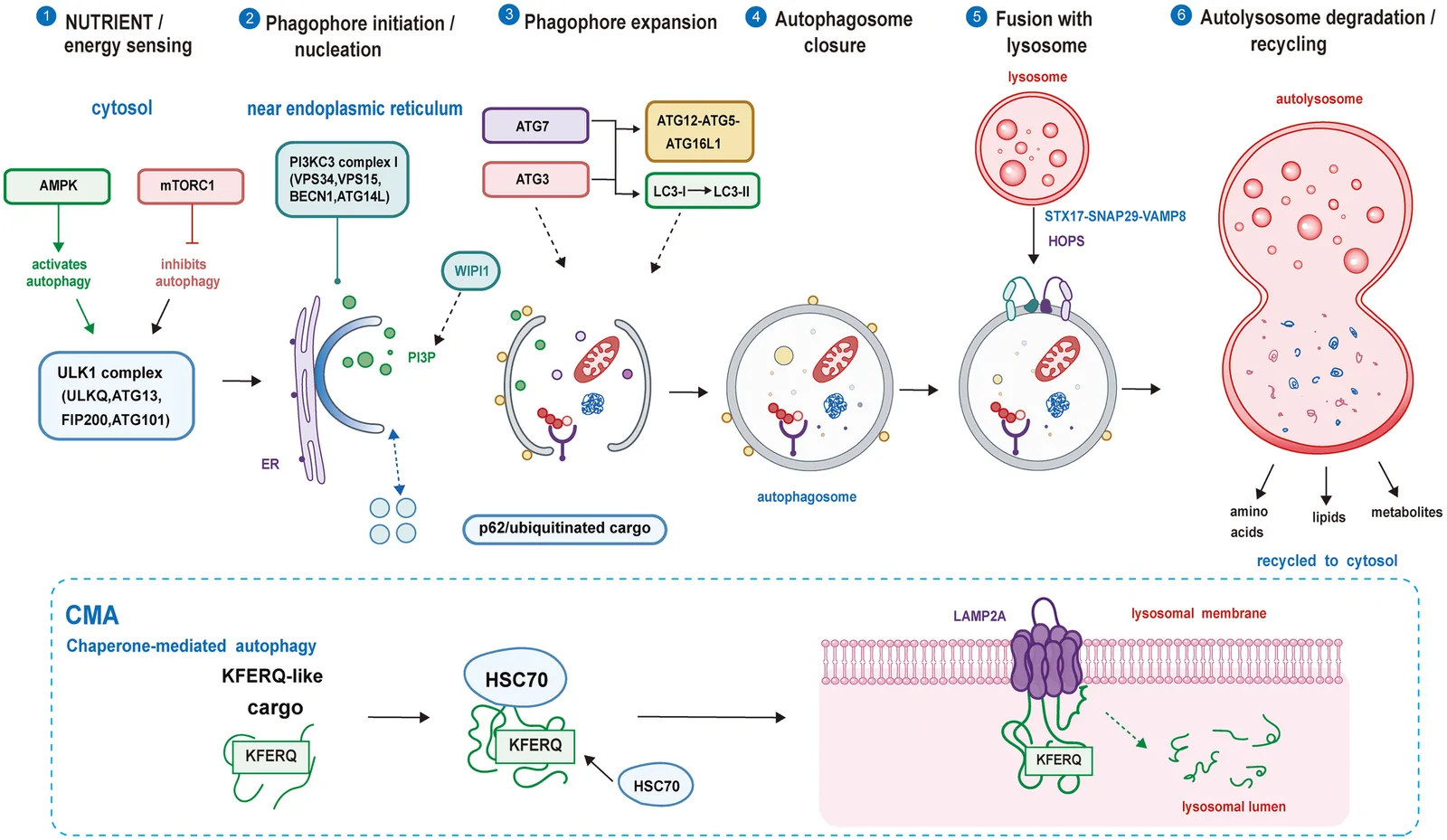
Molecular machinery and subcellular organization of the autophagy pathway. Nutrient and energy sensing by AMPK and mTORC1 regulates the ULK1 initiation complex in the cytosol. The PI3KC3 complex I generates PI3P at endoplasmic reticulum-associated phagophore assembly sites and recruits WIPI2, ATG9A-containing membranes and the ATG conjugation machinery. ATG12-ATG5-ATG16L1-dependent LC3/GABARAP lipidation promotes phagophore expansion, selective cargo recruitment and autophagosome closure. Mature autophagosomes fuse with lysosomes through HOPS and the STX17-SNAP29-VAMP8 SNARE machinery, generating autolysosomes for cargo degradation and metabolite recycling. The inset illustrates chaperone-mediated autophagy, in which HSC70 recognizes KFERQ-like substrates and delivers them to the lysosomal membrane receptor LAMP2A.

Autophagy is a highly conserved lysosome-dependent degradation and recycling mechanism in eukaryotic cells. Its primary function is to remove damaged organelles, abnormal protein aggregates and excess lipid components, thereby maintaining cellular homeostasis (62). According to the mode of substrate delivery, autophagy is commonly classified into macroautophagy, microautophagy and chaperone-mediated autophagy (CMA) (62–64). Macroautophagy is the most extensively studied form and proceeds through sequential stages of initiation, phagophore nucleation and expansion, autophagosome closure, autophagosome–lysosome fusion, and lysosomal degradation and recycling, as illustrated in Figure 2 (62, 65, 66).

Autophagy initiation is primarily regulated by the AMPK-mTORC1-ULK1 axis. Under nutrient-rich conditions, mTORC1 activity is high and suppresses ULK1 complex activation. During starvation, energy stress or oxidative stress, AMPK is activated and promotes autophagy initiation by inhibiting mTORC1 and directly regulating ULK1 (67, 68). Subsequently, the PI3KC3/VPS34 complex generates phosphatidylinositol 3-phosphate (PI3P), which recruits WIPI proteins and ATG-related complexes to promote phagophore nucleation and extension (69, 70). LC3/ATG8 lipidation contributes to autophagosome membrane maturation and cargo recognition (71). Finally, the autophagosome fuses with the lysosome to form the autolysosome, enabling substrate degradation and metabolic recycling (65, 72, 73).

At the molecular level, the genes involved in macroautophagy encode several functionally distinct classes of proteins. AMPK, mTOR and ULK1 are serine/threonine kinases, whereas ATG13, FIP200/RB1CC1 and ATG101 mainly function as regulatory or scaffold components of the ULK1 initiation complex. In the PI3KC3 complex I, VPS34 is a lipid kinase, VPS15 is a regulatory subunit, BECN1 functions as a scaffold protein and ATG14L directs the complex to autophagosome-forming membranes. PI3P subsequently recruits the *β*-propeller protein WIPI2, whereas the multipass transmembrane protein ATG9A contributes membrane components to the expanding phagophore. ATG7 and ATG3 function as E1- and E2-like enzymes, respectively, and the ATG12–ATG5–ATG16L1 complex provides E3-like activity for LC3/GABARAP lipidation. The selective autophagy receptor p62/SQSTM1 links ubiquitinated cargo to LC3-II on the phagophore membrane. Following autophagosome closure, tethering factors such as HOPS and SNARE proteins, including STX17, SNAP29 and VAMP8, mediate fusion with lysosomes, where acid hydrolases degrade the cargo and recycle the resulting metabolites (65, 66, 69–71).

Unlike macroautophagy, CMA does not require the formation of double-membrane autophagosomes (74). CMA mainly recognizes soluble substrate proteins containing KFERQ-like motifs and translocates them into the lysosomal lumen through the cytosolic molecular chaperone HSC70 and the lysosomal membrane receptor LAMP2A (63, 75). CMA participates in proteostasis, lipid metabolism, oxidative-stress responses and age-related homeostatic maintenance. Recent studies indicate that reduced CMA activity is associated with atherosclerosis progression, vascular wall cell stress and enhanced macrophage inflammation, suggesting that CMA may represent an important autophagic process linking metabolic dysfunction, ageing and CVDs (76–78).

### 3.2 Autophagy functions in cardiovascular cells

In vascular endothelial cells, moderate autophagy helps maintain endothelial barrier function, eNOS activity, mitochondrial quality control and redox balance (10, 11, 79, 80). Endothelial cells are chronically exposed to flow shear stress, oxidative stress, lipid overload and inflammatory stimulation, which can induce mitochondrial damage, ROS accumulation and proteostatic imbalance (81–83). By removing damaged mitochondria and abnormal proteins, autophagy reduces ROS generation, preserves NO bioavailability and limits the expression of inflammatory adhesion molecules, thereby protecting endothelial function (11, 84–86). Conversely, autophagy insufficiency leads to the accumulation of damaged mitochondria, enhanced oxidative stress and disruption of the endothelial barrier, whereas excessive autophagy activation or dysregulated autophagic flux may also cause cellular injury (87). Endothelial autophagy therefore exhibits intensity- and stage-dependent effects, and its protective role depends on whether autophagic flux is maintained within an appropriate range.

In macrophages, autophagy, particularly lipophagy, plays an important role in cholesterol handling and foam-cell formation (88). During atherosclerosis, macrophages engulf oxidized low-density lipoprotein and develop into foam cells. Autophagy delivers lipid droplets to lysosomes, where lysosomal acid lipase hydrolyses cholesteryl esters and provides free cholesterol for efflux, thereby limiting foam-cell formation and lipid core expansion (89). In advanced atherosclerosis, macrophage autophagy also protects plaque stability by restraining cell death, inflammation and necrotic core expansion (76, 90). When macrophage autophagy or CMA is impaired, lipid clearance declines, inflammasome activation and inflammatory cytokine release increase, and plaque progression and instability are promoted (76, 77, 90).

**Figure 3:**
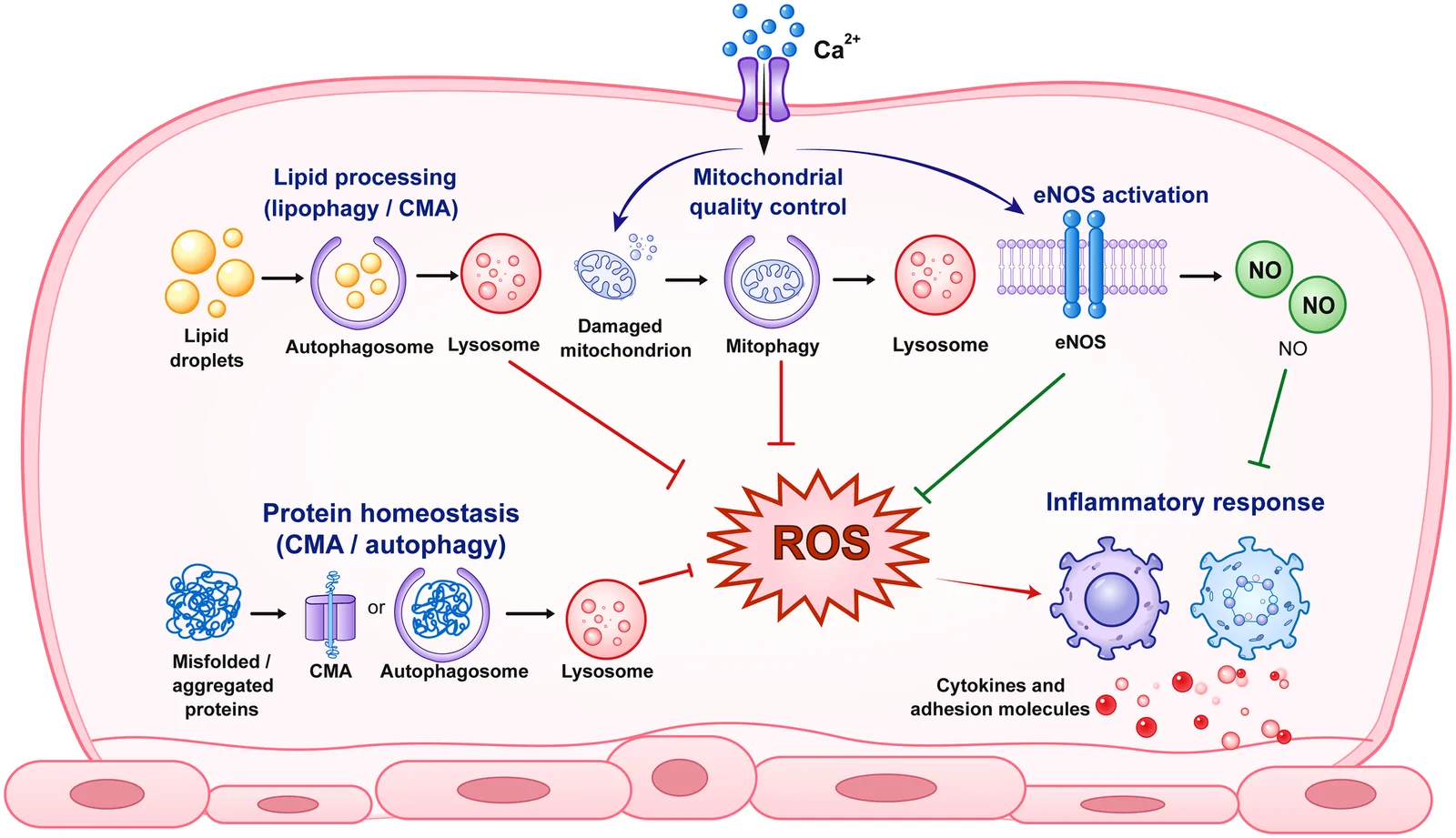
Autophagy-mediated maintenance of endothelial homeostasis. Autophagy contributes to vascular endothelial homeostasis by coordinating lipid processing, mitochondrial quality control, protein turnover, redox balance and inflammatory restraint. Lipophagy and CMA promote lipid droplet degradation and lipid recycling through autophagosome-lysosome pathways. Mitophagy removes damaged mitochondria and limits mitochondrial ROS production, whereas CMA and macroautophagy maintain proteostasis by degrading misfolded or aggregated proteins. Autophagy also supports eNOS activation and NO production. Impaired autophagic activity may lead to damaged organelle accumulation, excessive ROS generation, increased cytokine and adhesion molecule expression, and inflammatory activation.

In vascular smooth muscle cells (VSMCs), autophagy participates in phenotypic switching, matrix remodeling, calcification and regulation of fibrous-cap stability (91, 92). Moderate autophagy helps VSMCs cope with oxidative stress, lipid overload and endoplasmic reticulum stress and reduces abnormal calcium deposition. In contrast, impaired autophagic flux promotes VSMC senescence, death or functional dysregulation, leading to vascular wall damage and reduced plaque stability (91). In vascular calcification and aortic aneurysm, autophagy has context-dependent effects. Moderate activation may be protective, whereas excessive activation or flux blockade may aggravate pathological remodeling (91, 93). Therefore, VSMC autophagy should be evaluated in relation to disease stage, stimulus type and flux status rather than inferred from static markers such as LC3-II or p62 alone. LC3-II and p62 are steady-state markers rather than direct measurements of autophagic flux. LC3-II can accumulate either because autophagosome formation is increased or because autophagosome–lysosome fusion and lysosomal degradation are impaired. Conversely, reduced LC3-II levels may reflect either decreased autophagosome formation or accelerated lysosomal turnover. Similarly, p62 is degraded through autophagy but is also regulated at the transcriptional and translational levels by cellular stress and inflammatory signaling, meaning that its abundance may change independently of lysosomal degradation. Autophagic flux should therefore be assessed dynamically by comparing LC3-II or p62 levels in the presence and absence of lysosomal inhibitors, using tandem mCherry–GFP–LC3 reporters, or combining cargo-degradation assays with autophagosome–lysosome colocalization analysis. Whenever possible, multiple complementary approaches should be used rather than a single static marker (72, 73). In endothelial cells, autophagy contributes to vascular homeostasis by coordinating lipid processing, mitochondrial quality control, proteostasis, eNOS/NO signalling, ROS suppression and inflammatory restraint (Figure 3).

## 4 Primary cilia as upstream regulators of autophagic activity

Most mechanistic evidence that primary cilia regulate autophagy has been obtained from non-cardiovascular models, especially renal epithelial cells, embryonic stem cells and fibroblast systems. Therefore, the following pathways should be interpreted as biologically plausible regulatory modules rather than fully established cardiovascular pathways unless direct vascular evidence is available.

Within this evidence boundary, early studies linked primary cilia to Hedgehog (Hh) signaling and autophagy initiation (94). Pampliega et al. showed that, under starvation, primary cilia can promote autophagy initiation through Hh signaling and IFT-related transport; several ATG proteins can be recruited to the ciliary base, suggesting that the ciliary base may serve as a platform for assembling autophagy-related complexes and integrating local signals (21). This work established a functional connection between primary cilia and autophagy, but because the evidence was not primarily derived from cardiovascular cells, it should be cited as foundational mechanistic evidence rather than as proof of a cardiovascular-specific pathway.

In addition to ligand-dependent signals, mechanical stimulation is an important upstream factor linking primary cilia to autophagy (16, 95). In renal epithelial cells, fluid shear stress can suppress mTORC1 activity through primary cilia and engage LKB1-AMPK-related signaling (96). Orhon et al. further demonstrated that fluid shear stress induces primary cilium-dependent autophagy and contributes to epithelial volume adaptation (97). Zemirli et al. reported that the ciliary protein folliculin (FLCN) participates in LKB1 recruitment to the ciliary region and affects AMPK activation, ATG16L1 recruitment and shear stress-induced autophagy (98). These studies support a mechanosensing-autophagy coupling mechanism in epithelial models; whether a comparable pathway operates in vascular endothelium under disturbed or low shear stress remains to be directly tested.

The polycystin complex is another important node in ciliary regulation of autophagy (99). PC1/PC2 are classical cilia-associated mechanosensory proteins and participate in mechanically induced Ca^2+^ signal transduction. In vascular endothelial cells, PC1/PC2 are closely linked to shear-stress sensing, Ca^2+^ signaling, eNOS activity and NO generation (8, 100). In the context of autophagy, PKD2/PC2 has been reported to form a complex with BECN1 and to participate in autophagy induction through Ca^2+^-related mechanisms (100, 101). However, direct evidence for this mechanism in cardiovascular cells, particularly vascular endothelial cells, remains limited.

At the membrane-lipid signaling level, PI3KC2*α*-dependent PI3P generation is considered an important mechanism by which primary cilia regulate autophagy (28, 102). In renal epithelial cells, shear stress-induced autophagy does not entirely depend on the canonical VPS34/PI3KC3 pathway. Instead, class II PI3K, PI3KC2*α*, generates a local pool of PI3P near the primary cilium and promotes the formation of autophagy-related membrane structures; PI3KC2*α* deficiency attenuates shear-induced PI3P production, autophagic responses and cell volume adaptation (28). ATG16L1 also cooperates with IFT20 and INPP5E to regulate ciliary phosphoinositide metabolism, suggesting spatial coupling among autophagy proteins, ciliary transport systems and membrane-lipid signaling (27). This mechanism is particularly relevant to vascular disease because low shear stress and disturbed flow are characteristic features of atherosclerosis-prone regions. Importantly, endothelial studies show that a PI3KCIIalpha-dependent autophagy program protects endothelial function and limits atherosclerosis progression under low shear stress (103). However, whether endothelial primary cilia directly coordinate PI3KC2*α*-dependent autophagy in these regions remains incompletely established.

Primary cilia may also influence autophagy through redox stress and transcriptional regulatory networks (25). Jang et al. proposed the primary cilium-autophagy-NRF2 (PAN) axis, in which primary cilia regulate NRF2 activity through autophagic flux and participate in neuroectodermal differentiation of human embryonic stem cells (104). Subsequent studies found that NRF2 activity is increased in cilia-deficient fibroblasts and that mTOR inhibitors can partially reverse this phenotype by enhancing autophagy (105). These findings suggest that cilium-autophagy crosstalk may regulate cellular oxidative-stress responses. Given that ROS accumulation is a common pathological feature of endothelial injury, atherosclerosis and aortic aneurysm (106), the PAN axis may have potential cardiovascular relevance. However, this mechanism has mainly been established in hESCs and fibroblasts and has not yet been validated in the cardiovascular system.

Taken together, available studies support the capacity of primary cilia to modulate autophagy in response to nutrient deprivation or mechanical stress, but they do not identify a single universal upstream pathway. Hh/IFT-dependent recruitment, LKB1–AMPK–mTOR signaling, PI3KC2*α*-derived PI3P, PC2/Ca^2+^ signaling and NRF2 regulation may represent context-dependent and potentially complementary modules rather than mutually exclusive mechanisms. The strongest causal evidence currently comes from renal epithelial models. Although endothelial studies support a protective PI3KC2*α*-dependent autophagy program under low shear stress, they have not yet demonstrated that this response is directly dependent on primary cilia. The principal unresolved question is therefore whether ciliary disruption alters dynamic autophagic flux and vascular phenotype within the same cardiovascular model.

## 5 Autophagy as a regulator of ciliogenesis and ciliary stability

Evidence that autophagy regulates ciliogenesis and ciliary stability is robust in several non-cardiovascular models, but its relevance to vascular endothelial cells and VSMCs remains largely untested. The mechanisms below should therefore be read as a framework for potential vascular regulation rather than as confirmed cardiovascular pathways.

Autophagy is not only regulated by primary cilia but also reciprocally influences ciliogenesis, ciliary length maintenance and ciliary functional homeostasis (107). Pampliega et al. showed that basal autophagy can degrade the ciliogenesis-related protein IFT20, thereby limiting excessive cilium formation (21). Tang et al. further demonstrated that starvation-induced autophagy selectively removes the ciliogenesis inhibitor OFD1 from centriolar satellites, relieving its inhibitory effect and promoting primary cilium formation (22). These findings indicate that autophagy does not simply promote or inhibit ciliogenesis; rather, it dynamically regulates ciliary homeostasis according to nutrient status, substrate identity and cellular context (21–23, 108, 109). Whether analogous OFD1- or IFT20-dependent regulation controls endothelial or VSMC ciliary length and mechanosensing under cardiovascular stress remains unclear.

Autophagy-related proteins can also participate in ciliary protein transport and ciliary membrane construction through non-canonical mechanisms (107–109). ATG16L1 not only participates in autophagosome formation but also cooperates with IFT20 and INPP5E to regulate ciliary phosphoinositide metabolism and ciliary protein transport. IFT20 can also recruit ATG16L1 to Golgi- and early endosome-associated membrane structures (27, 110). These findings indicate that autophagy-related molecules are not restricted to lysosomal degradation pathways but may also help maintain ciliary membrane composition, protein delivery and local lipid homeostasis. In the cardiovascular system, if similar mechanisms operate in endothelial cells, autophagy dysfunction may impair IFT20 transport, ciliary membrane lipid composition and receptor localization, thereby weakening endothelial shear-stress sensing.

Under pathological conditions, autophagy dysfunction may also disrupt ciliary homeostasis (109). Studies in Huntington's disease have linked abnormal protein aggregation and impaired autophagy-lysosome function with structural abnormalities of primary cilia (111). This observation suggests that, in disease environments characterized by proteostatic disruption, autophagy abnormalities may affect ciliary protein renewal and ciliary structural maintenance. The selective degradation of ciliary components is termed ciliophagy. Lam et al. reported in COPD-related models that cigarette smoke induces autophagy enhancement and ciliary shortening, and that HDAC6-mediated selective autophagy contributes to ciliary dysfunction (112). Although these findings mainly derive from neurological or respiratory disease models, they suggest that in chronic inflammation, oxidative stress and proteostatic imbalance, abnormal autophagy may aggravate cellular sensing dysfunction by affecting ciliary component degradation or protein transport.

Crosstalk between autophagy and the ubiquitin-proteasome system (UPS) also contributes to ciliary homeostasis. Wang et al. reported reciprocal regulation between cilia and autophagy through the mTOR and proteasome pathways, suggesting that ciliary homeostasis does not rely on a single degradative pathway (113). The ciliary transition-zone protein RPGRIP1L has also been linked to autophagy and proteasome function. Struchtrup et al. found that RPGRIP1L regulates autophagy independently of its function in controlling proteasomal activity at the ciliary base (114). Gerhardt et al. further reported that RPGRIP1L regulates proteasomal activity at the ciliary base through Psmd2, thereby affecting the clearance of ciliary signaling proteins (115). Thus, ciliary homeostasis is more likely maintained by the coordinated actions of autophagy, UPS, IFT transport and membrane-lipid signaling than by a single pathway.

**Figure 4:**
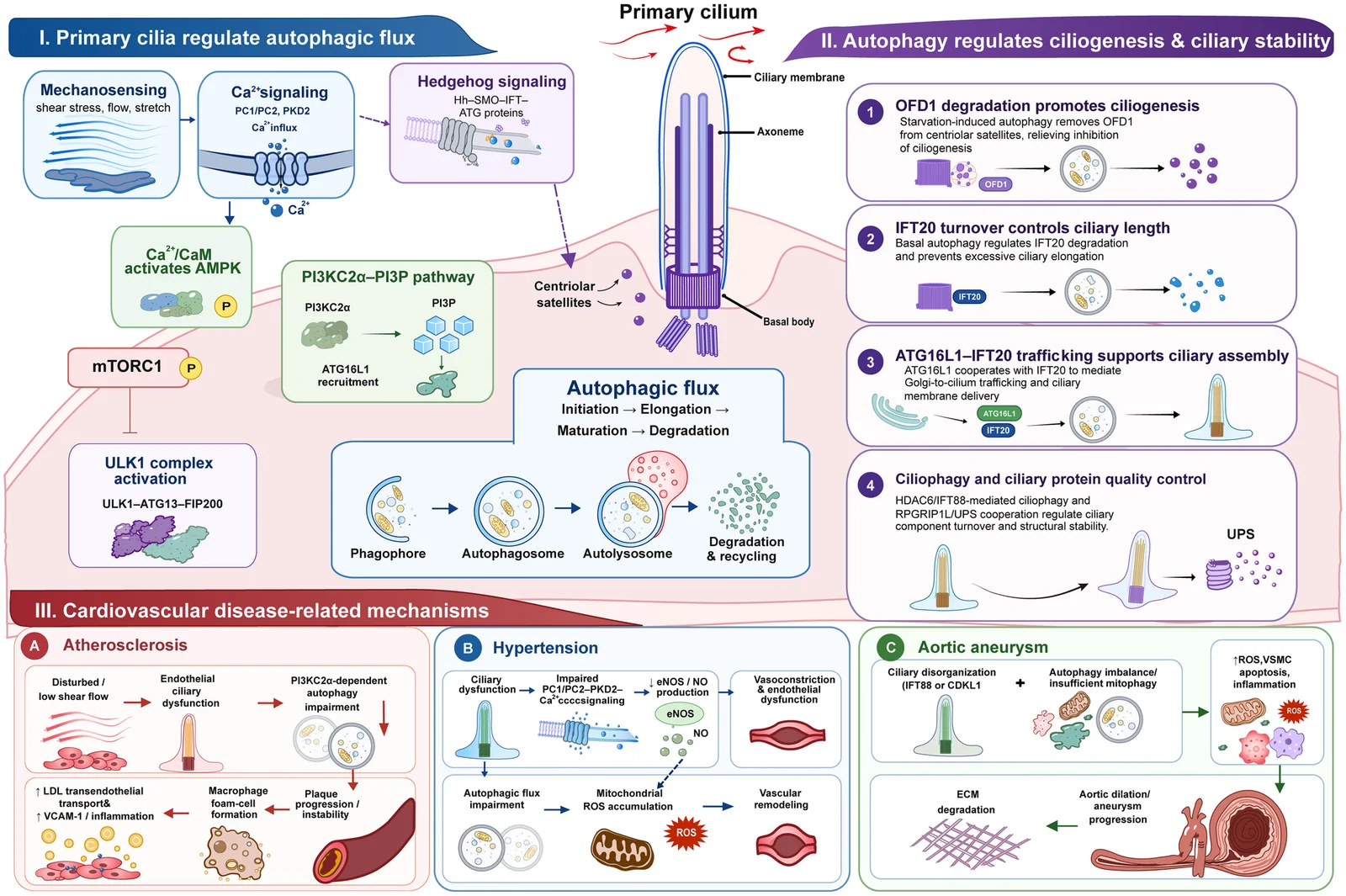
Bidirectional crosstalk between primary cilia and autophagy in cardiovascular disease. Primary cilia may regulate autophagic flux through shear-stress mechanosensing, Ca2+/calmodulin-mediated AMPK activation, mTORC1 inhibition, ULK1 complex activation, Hedgehog signaling and the PI3KC2*α*-PI3P pathway. Autophagy reciprocally regulates ciliogenesis and ciliary stability through OFD1 removal, IFT20 turnover, ATG16L1-IFT20-mediated trafficking, ciliophagy and ciliary protein quality control involving HDAC6, IFT88, RPGRIP1L and the UPS. Disease-related mechanisms include impaired endothelial ciliary function and PI3KC2*α*-dependent autophagy in atherosclerosis, reduced PC1/PC2-PKD2-Ca2+-eNOS/NO signaling and mitochondrial ROS accumulation in hypertension, and ciliary disorganization together with autophagy imbalance or insufficient mitophagy in aortic aneurysm.

Collectively, the available studies agree that autophagy contributes to ciliary homeostasis through selective protein turnover, membrane trafficking and quality control, but the direction of its effect is strongly dependent on the substrate and cellular context. Starvation-induced degradation of OFD1 promotes ciliogenesis, whereas basal turnover of IFT20 may restrain excessive ciliary growth, and stress-induced ciliophagy can contribute to ciliary shortening or dysfunction. These apparently different outcomes are not necessarily contradictory; rather, they suggest that autophagy maintains an optimal ciliary state instead of uniformly promoting or suppressing ciliogenesis. However, this principle has not been directly tested in vascular endothelial cells or VSMCs, and it remains unknown whether manipulating autophagic flux can restore ciliary mechanosensing under vascular stress. Because most mechanistic evidence for cilium–autophagy crosstalk is derived from non-cardiovascular models, the direction of regulation, experimental context, evidence level and cardiovascular relevance of each mechanism are summarized in Table 1. Disease-specific relevance of the primary cilium-autophagy interface in vascular diseaseBased on the mechanisms described above, primary cilia and autophagy may contribute to cardiovascular disease progression by affecting endothelial mechanosensing, autophagic flux, mitochondrial quality control, ROS homeostasis, NO production, lipid handling and inflammatory responses. Taken together, these findings support a working model in which primary cilia and autophagy form a bidirectional regulatory network that may influence vascular pathology across hypertension, atherosclerosis and aortic aneurysm (Figure 4). However, the strength of disease-specific evidence differs substantially across vascular disorders. Among the available evidence, atherosclerosis is relatively well supported, whereas hypertension and aortic aneurysm are more dependent on independent observations of ciliary dysfunction or autophagy dysregulation. Across these vascular disorders, the literature consistently indicates that ciliary dysfunction and autophagy imbalance can each contribute to vascular injury. However, the evidence that they form a directly coupled pathway is uneven. Atherosclerosis provides the strongest convergence because both endothelial cilia and endothelial autophagy respond to disturbed or low shear stress. In hypertension and aortic aneurysm, the two processes are mainly connected through shared signaling nodes or parallel pathological observations. The central unresolved issue is whether AMPK/mTOR, Ca^2+^ and ROS represent direct mediators of cilium-autophagy crosstalk or common downstream responses to vascular stress. The definitions of evidence levels A-C, together with the disease-specific mechanisms and unresolved gaps, are summarized in Table 2 Hypertension.

**Table 1:** Mechanistic evidence for bidirectional crosstalk between primary cilia and autophagy.

Direction	Mechanistic module	Experimental model	Key finding	Key references	Evidence level	Cardiovascular relevance/key gap
Primary cilia regulate autophagy	Hh-dependent autophagy initiation	Starvation models; MEFs and other non-cardiovascular cells	Hh signaling promotes autophagy in an IFT-dependent manner; ATG proteins may localize near the ciliary base, suggesting a ciliary platform for autophagy initiation.	(21, 116)	B	Direct validation in endothelial cells or VSMCs is lacking.
Primary cilia regulate autophagy	Shear stress-dependent ciliary autophagy	Renal epithelial flow models	Fluid shear stress activates cilium-dependent LKB1-AMPK signaling, suppresses mTORC1 and promotes ULK1-related autophagic flux; FLCN helps link ciliary mechanosensing to AMPK-dependent autophagy.	(96–98)	B	Supported in epithelial models; vascular validation under disturbed or low shear stress is needed.
Primary cilia regulate autophagy	PI3KC2*α*-PI3P lipid signaling	Renal epithelial shear-stress models	PI3KC2*α* generates local PI3P near primary cilia and drives shear stress-induced, VPS34-independent autophagy and autophagosome formation.	(28)	B	Endothelial cilium-dependent PI3KC2*α* autophagy in lesion-prone flow regions remains incompletely established.
Primary cilia regulate autophagy	PAN axis and oxidative-stress response	hESC and MEF models	The primary cilium-autophagy-NRF2 axis links ciliary status with autophagic activity, p62/KEAP1/NRF2 signaling and oxidative-stress responses.	(104)	B/C	Vascular ROS relevance remains largely inferential.
Primary cilia regulate autophagy	Polycystin/Ca^2+^-related mechanosensing	Endothelial and renal mechanosensing models	PC1/PC2 and PKD2 mediate ciliary Ca^2+^ signaling and endothelial NO generation; direct control of autophagic activity in cardiovascular cells remains unproven.	(8, 52, 100, 101)	B/C	A complete PC1/PC2-Ca^2+^-autophagy causal chain in vascular disease has not been demonstrated.
Autophagy regulates cilia	OFD1 degradation and ciliogenesis	Non-cardiovascular mammalian cell models	Starvation-induced autophagy removes OFD1 from centriolar satellites, relieving inhibition of ciliogenesis.	(22)	B	Endothelial or VSMC validation under cardiovascular stress is lacking.
Autophagy regulates cilia	IFT20 turnover and ciliary length control	Non-cardiovascular cell models	Basal autophagy regulates IFT20 turnover and limits excessive ciliary elongation.	(21, 22)	B	Direct vascular evidence for IFT20 turnover controlling ciliary mechanosensing is insufficient.
Autophagy regulates cilia	ATG16L1-IFT20 trafficking and ciliary membrane assembly	Ciliary trafficking and renal epithelial-related models	ATG16L1 cooperates with IFT20 and INPP5E to regulate phosphoinositide turnover, Golgi-to-cilium trafficking and ciliary dynamics.	(27)	B	Its effect on endothelial shear-stress sensing remains unknown.
Autophagy regulates cilia	Ciliophagy and ciliary component degradation	Airway epithelial/COPD-related models	HDAC6-mediated selective autophagy promotes ciliary component turnover and contributes to ciliary shortening or dysfunction.	(112)	B/C	Evidence mainly comes from airway models; vascular relevance requires testing.
Autophagy regulates cilia	RPGRIP1L/UPS-dependent ciliary quality control	MEFs and ciliopathy models	RPGRIP1L links autophagy, mTORC1 and proteasome-related ciliary quality control at the ciliary base.	(114, 115)	B/C	Cardiovascular relevance remains speculative.

Evidence level: Level A, direct evidence from cardiovascular cells, vascular tissues or cardiovascular disease animal models with relatively clear functional relevance; Level B, evidence mainly from non-cardiovascular models but with clear mechanistic relevance to vascular biology, or cardiovascular evidence supporting only one side of the primary cilium-autophagy interface; Level C, indirect evidence, theoretical inference or separate evidence for primary cilia and autophagy without direct causal crosstalk.

**Table 2:** Disease-specific evidence for the primary cilium-autophagy interface in vascular disease.

Disease/process	Major cell type/model	Primary cilium-related evidence	Autophagy-related evidence	Proposed convergent node	Key references	level	Key gap
Hypertension	Endothelial cells; VSMCs; ciliary cardiovascular risk models	Evidence mainly supports disruption of the PC1/PC2-PKD2-Ca^2+^-eNOS-NO axis, DR5-related ciliary mechanosensing and BBSome-related blood pressure regulation.	Autophagy may contribute through mitochondrial ROS, endothelial dysfunction and vascular remodeling, but the cilium-autophagy link is not established.	PC1/PC2-PKD2-Ca^2+^-eNOS-NO; AMPK-mTORC1; mitochondrial ROS	(8, 51, 52, 100, 118)	B/C	Direct evidence for a cilium-autophagic activity-hypertension causal chain is lacking.
Atherosclerosis—endothelial cells	Endothelial cells; ApoE-deficient mouse models; low shear-stress endothelial models	Endothelial cilia are enriched or altered in disturbed-flow regions; loss of endothelial cilia promotes endothelial inflammation and atherosclerosis.	PI3KC2*α*-dependent endothelial autophagy protects against low shear stress-induced endothelial dysfunction and atherosclerosis.	Disturbed flow-endothelial cilia-PI3KC2*α*-PI3P-autophagy; LDL transport; VCAM-1/inflammation	(7, 9, 43, 45, 103, 134)	A/B	The direct *in vivo* link between endothelial cilia and PI3KC2*α*-dependent autophagy needs validation.
Atherosclerosis—macrophages	Macrophage foam cells; mouse and human atherosclerotic models	Direct evidence for macrophage primary cilia is limited.	Macrophage autophagy, lipophagy and CMA regulate lipid droplet degradation, cholesterol efflux, inflammation and foam-cell formation.	Autophagy-lysosome pathway; lipophagy; LAMP2A-dependent CMA; cholesterol efflux	(77, 89, 123)	B/C	Whether macrophage cilia participate in foam-cell formation or interact with lipophagy/CMA remains unclear.
Atherosclerosis—VSMCs	VSMCs; atherosclerotic plaque models	Direct evidence linking VSMC ciliary changes to plaque progression is limited.	VSMC autophagy participates in phenotypic switching, apoptosis, senescence, calcification and plaque stability.	AMPK-mTOR; ROS; mitophagy; ER stress; VSMC phenotypic switching	(91, 126)	B/C	The cilium-autophagy interaction in VSMCs during plaque progression remains poorly defined.
Aortic aneurysm	Endothelial cells; VSMCs; aortic aneurysm models	Recent evidence links CDKL1-related ciliary defects with thoracic aortic aneurysm; ciliary disorganization may impair vascular mechanosensing.	Aneurysm studies suggest VSMC loss, ECM remodeling, ROS accumulation, autophagy imbalance and defective mitophagy.	Mechanosensing-Ca^2+^/AMPK-mTOR; ROS; mitophagy; ECM degradation	(53, 91, 127, 132, 135)	C	Whether ciliary abnormalities and autophagy imbalance form a causal crosstalk axis remains unproven.

Evidence level: Level A, direct evidence from cardiovascular cells, vascular tissues or cardiovascular disease animal models with relatively clear functional relevance; Level B, evidence mainly from non-cardiovascular models but with clear mechanistic relevance to vascular biology, or cardiovascular evidence supporting only one side of the primary cilium-autophagy interface; Level C, indirect evidence, theoretical inference or separate evidence for primary cilia and autophagy without direct causal crosstalk.

Hypertension is closely associated with abnormal vascular tone, endothelial dysfunction, enhanced oxidative stress and vascular remodeling. Current cilia-related evidence in hypertension centers on the endothelial PC1/PC2–PKD2 mechanosensory complex, whose disruption may impair Ca^2+^-dependent eNOS activation, NO production and vasodilation (8, 117). DR5 has also been reported to localize to primary cilia and participate in chemical and mechanical sensing, whereas BBSome abnormalities are associated with obesity, hypertension and metabolic cardiovascular risk phenotypes (51, 52, 118).

Autophagy-related evidence in hypertension primarily concerns mitochondrial quality control and redox homeostasis. Impaired autophagic flux may promote mitochondrial ROS accumulation and endothelial or VSMC dysfunction, thereby contributing to abnormal vascular tone and remodeling (10, 11, 119, 120). At present, however, the ciliary and autophagy evidence remains largely parallel rather than causally integrated. Primary ciliary defects may plausibly impair endothelial flow sensing and influence AMPK/mTOR-related autophagy regulation based on non-cardiovascular mechanosensing models (96–98), whereas autophagy dysfunction may aggravate endothelial mechanosensing abnormalities through ciliary protein turnover or mitochondrial ROS accumulation (119). Direct evidence for a complete cilium-autophagic flux-hypertension causal chain is still lacking. Accordingly, the hypertension evidence should be interpreted as Level B/C rather than as direct proof of a cardiovascular cilium-autophag*y* axis.

### 5.1 Atherosclerosis

Atherosclerosis is currently one of the cardiovascular diseases most closely linked to research on primary cilia and autophagy (103). In endothelial cells, primary cilia show a clear flow-dependent distribution (9). Regions of low shear stress, oscillatory shear stress or disturbed flow, such as arterial branches and curvatures, are atherosclerosis-prone sites and are also regions in which endothelial cilia are relatively enriched (6). Endothelial cilia mediate Ca^2+^ signaling through PC1/PC2 and regulate eNOS activity and NO production, thereby contributing to vasodilation and endothelial anti-inflammatory responses (8, 121). When ciliary genes such as IFT88 are disrupted, endothelial mechanosensing declines, eNOS activity decreases, inflammation-related gene expression increases and atherosclerosis is promoted under high-fat dietary conditions (43). Ciliary loss can also enhance shear-induced EndMT-related responses, indicating a potential role in linking abnormal flow to vascular pathological remodeling. At the same time, endothelial autophagy in low-shear regions has attracted attention (122). A PI3KCIIalpha-dependent autophagy program protects endothelial function and limits atherosclerosis progression under low shear stress (103). Together with the PI3KC2*α*-PI3P mechanism described above, PI3KC2*α*-related membrane-lipid signaling may represent an important node connecting low shear stress, ciliary mechanosensing and autophagic flux (28, 103).

In macrophages, autophagy mainly influences foam-cell formation and plaque stability through lipophagy and CMA (123). After uptake of oxidized low-density lipoprotein, macrophages accumulate lipid droplets and cholesterol. An intact autophagy-lysosome pathway promotes lipid droplet degradation, cholesterol efflux and lysosomal lipid handling, thereby limiting foam-cell formation and lipid core expansion (89). In advanced atherosclerosis, macrophage autophagy also restrains cell death, inflammation and necrotic core expansion, thereby contributing to plaque stability (78, 90). CMA mediates LAMP2A-dependent degradation of specific substrate proteins and participates in lipid metabolism and inflammatory regulation; its dysfunction is associated with enhanced inflammation and atherosclerosis progression (76, 77, 124, 125). Although the role of primary cilia in macrophages is much less defined than that of endothelial cilia, macrophage autophagy dysfunction can alter the plaque microenvironment through lipid accumulation, ROS elevation and inflammatory amplification, thereby indirectly aggravating endothelial injury and plaque progression.

In VSMCs, autophagy participates in phenotypic switching, calcification, cell death and regulation of fibrous-cap stability (92). Moderate autophagy helps maintain VSMC homeostasis and reduce oxidative stress, whereas autophagy deficiency promotes VSMC senescence, death and decreased plaque stability (13, 91). VSMC autophagy dysfunction may also aggravate cellular damage and plaque complexity in atherosclerosis (126). Among the three major vascular cell types involved in atherosclerosis, endothelial cells currently provide the most plausible site for direct primary cilium-autophagy crosstalk because endothelial cilia are flow-sensitive and endothelial autophagy is regulated by low shear stress (28, 103). In macrophages and VSMCs, autophagy clearly regulates lipid handling, inflammation, phenotypic switching and plaque stability, but direct evidence that these processes are controlled by primary cilia remains limited (91, 126). Thus, the apparent convergence at the whole-plaque level should not be interpreted as proof of a cilium-dependent autophagy pathway in every vascular cell type.

### 5.2 Aortic aneurysm

Aortic aneurysm development involves hemodynamic abnormalities, VSMC apoptosis or phenotypic switching, elastic fiber degradation, inflammatory infiltration, oxidative stress and extracellular matrix remodeling (127). In recent years, ciliary molecules have begun to attract attention in the context of aortic wall structure and vascular wall cell mechanosensing. VSMCs can form primary cilia and may use them to sense extracellular matrix and mechanical stimuli (53, 128). Recent genetic evidence indicates that CDKL1 variants affect ciliogenesis and are associated with susceptibility to thoracic aortic aneurysm; CDKL1 expression has also been observed in VSMCs of normal and diseased human aortic walls (53). These findings suggest that abnormal ciliogenesis may contribute to impaired mechanosensing and structural homeostasis of aortic wall cells, although the evidence remains at an early stage.

Autophagy also has dual roles in aortic aneurysm (129, 130). In aortic aneurysm models, preserved autophagic and mitophagic flux has been associated with reduced mitochondrial damage, ROS accumulation and VSMC loss, whereas impaired flux accompanies vascular wall degeneration and aneurysmal remodeling (91, 131). In aortic aneurysm models, VSMC autophagy has been suggested to help preserve aortic wall integrity and reduce aneurysm severity (132, 133). These findings support a potentially protective role for intact autophagic quality control, although the consequences may vary according to disease stage and experimental context.

At present, direct evidence for primary cilium-autophagy crosstalk in aortic aneurysm remains weak. Existing studies separately support the involvement of abnormal ciliogenesis and autophagy imbalance in aneurysm pathogenesis, but they do not prove a causal interaction through a shared mechanosensing, ROS or mitochondrial quality-control axis. A more cautious interpretation is that hemodynamic abnormalities or AngII stimulation may impair cilia-related mechanosensing in endothelial cells and VSMCs and alter Ca^2+^/AMPK/mTOR signaling, while abnormal autophagic activity may cause mitochondrial injury, ROS accumulation and enhanced inflammation, thereby promoting matrix degradation and vascular wall remodeling. This proposed convergence should be tested using cell-specific ciliary-defect models, dynamic autophagic flux assays and human aortic tissue validation.

## 6 Conclusions and perspectives

Primary cilia and autophagy represent complementary systems for extracellular sensing and intracellular quality control. Their functional coupling is most plausible in flow-responsive vascular endothelial cells, but the strength of evidence remains highly dependent on disease context and cell type. Atherosclerosis currently provides the most coherent support for a vascular primary cilium–autophagy interface, whereas hypertension and aortic aneurysm rely more heavily on mechanistic extrapolation and parallel observations of ciliary dysfunction and autophagy imbalance. Accordingly, the available literature supports the primary cilium-autophag*y* axis as a useful mechanistic framework, but not yet as a universally established causal pathway across vascular diseases or vascular cell types.

Several limitations remain in the current literature. First, much of the mechanistic evidence comes from renal epithelial cells, embryonic stem cells, neurological models or respiratory models and cannot be directly equated with processes in cardiovascular cells. Second, some studies still evaluate autophagy using static markers such as LC3-II and p62, rather than systematically assessing dynamic autophagic flux. Third, mechanisms may differ substantially across vascular cell types. Endothelial cells, macrophages and VSMCs have distinct functions in vascular pathology, and cilia- and autophagy-related mechanisms should not be indiscriminately extrapolated among them. Fourth, direct evidence from human vascular tissues and clinical disease samples remains insufficient, limiting the assessment of this axis as a therapeutic target.

Future studies should focus on testing the causal chain of ciliary alteration-autophagic activity-vascular pathological phenotype. Endothelial- or VSMC-specific ciliary-defect models should be combined with mCherry-GFP-LC3, lysosomal inhibitors and mitophagy reporter systems to dynamically assess autophagic flux. Under high-fat, AngII, high-salt, low-shear and oxidative-stress conditions, ciliary length, IFT protein localization, PC1/PC2 distribution, p-AMPK/p-mTOR signaling, mitochondrial ROS and NO generation should be measured in parallel. Combined colocalization analyses in human atherosclerotic plaques and aortic aneurysm tissues may further clarify the causal role and potential interventional value of the primary cilium-autophagy interface in vascular disease. Whether comparable primary cilium-autophagy crosstalk occurs in lymphatic endothelial cells also warrants further investigation.
